# Retrospective analysis of adaptation frequencies and factors in offline in offline adaptive proton therapy

**DOI:** 10.1002/acm2.70604

**Published:** 2026-05-27

**Authors:** Farideh Pak, Hailei Zhang, Yao Hao, Matthew Schmidt, Weiren Liu, Joshua Schiff, Eric Laugeman, Michael Watts, Stephanie Perkins, Tianyu Zhao

**Affiliations:** ^1^ Department of Radiation Oncology School of Medicine Washington University in St. Louis Saint Louis Missouri USA; ^2^ Department of Radiation Oncology Proton & Advanced Radiation Center Atrium Health Levine Cancer Institute Charlotte North Carolina USA; ^3^ Keck School of Medicine of USC Department of Radiation Oncology CA Florida USA; ^4^ Department of Radiation Oncology University of South Florida Tampa Florida USA

**Keywords:** adaptive, breast and chest wall, head and neck, prostate, proton therapy

## Abstract

**Purpose/objective:**

Offline adaptation is a standard practice in radiation therapy to adjust treatment plans for various reasons. Although offline adaptation in proton therapy has been addressed in prior studies, further research is still needed to clarify the underlying reasons. Because of the unique dosimetric properties of protons, it is essential to know why and when offline adaptation is needed. In this retrospective study, in‐room CT‐on‐Rails (CToR) scans were used to characterize the reasons and frequency of offline adaptive proton therapy for head and neck (HN), breast or chest wall (breast/CW), and prostate cases.

**Materials/methods:**

A cohort of 166 cases receiving proton therapy from 2020 to 2023 were reviewed retrospectively, including 76 HN cases, 50 breast/CW, and 40 prostate cases. The imaging frequency and incidence of replanning were recorded, and the rationales for replanning were investigated through anatomic review, dose volume histogram (DVH) comparison, and dose evaluations.

**Results:**

Overall, replanning occurred in 11% of all the cases, with 13% of HN and 16% of breast/CW cases needing replanning. No prostate cases required replanning. HN cases were planned in 20 to 33 fractions, and no timing pattern of replanning emerged. Breast/CW were planned in 16 to 25 fractions, with replanning peaking at fractions 3–6 and 14 and again at 17. Anatomy changes most frequently triggered offline adaptation for HN cases, while more offline adaptation in breast/CW cases were due to displacement of tissue expander magnets.

**Conclusion:**

This study uncovered different trends in the rationale for offline adaptation in proton therapy. HN cancer patients were more likely to need adaptation due to anatomy changes whereas breast/CW patients more often needed adaptation due to tissue expander magnet migration. Prostate cases showed stable treatment plans and dose distribution through the treatment course.

## 1 INTRODUCTION

The concept of adaptation in radiotherapy allows for tailoring individual radiation treatment plans to account for the dynamic nature of tumors and patient anatomy.^1^ Similar to conventional photon radiotherapy, proton therapy most commonly utilizes offline adaptive approaches to optimize treatment precision and patient outcomes.^2^ Typically, this process is triggered by daily imaging revealing anatomical changes, potentially affecting the radiation dose to the target and surrounding tissues. Consequently, the treatment plan may need to be re‐simulated and adjusted.^3^, ^4^, ^5^, ^6^ However, effectively managing the daily anatomical and tissue density variations remains a significant challenge. These fluctuations have the potential to impact the precision of proton radiation delivery and, consequently, treatment outcomes.

Because of the unique dosimetric properties of proton therapy, understanding the frequency and motivations for implementing offline adaptive proton is vital to improving treatment predictability, plan optimization, and delivery of quality and efficient treatment.^2^ Previous studies evaluated specific scenarios of when offline adaptations in proton therapy occurred or focused on a single disease site. Recent research by Uh et al. recommended on‐treatment imaging three weeks after simulation for pediatric patients with para‐meningeal rhabdomyosarcoma undergoing upfront proton therapy, especially when simulation occurs one week after chemotherapy initiation.^7^ Ger et al. proposed criteria for robustness, daily imaging, and repeated computed tomography (CT) frequency in proton therapy for breast cancer patients, suggesting daily cone beam CT for setup uncertainties and repeated QA CTs for patients with expanders or implants.^8^ Huiskes et al. investigated the impact of adaptive proton therapy (APT) on dose distribution and optimal timing for plan adaptation in head and neck cancer, finding that a single adaptive intervention significantly improved target coverage, with subsequent interventions further enhancing coverage while minimizing organ‐at‐risk (OAR) doses, although the optimal timing remains uncertain.^9^

Although offline adaptation in proton therapy has been addressed in prior studies, further investigation could provide valuable insights. In this study, we aim to provide a thorough understanding of the factors driving offline adaptations across diverse anatomical positions by exploring a range of common cancer types like HN, breast or chest wall (breast/CW) and prostate, and by extracting the frequency of these adaptations. These findings could improve treatment precision and effectiveness, potentially aiding in the identification of situations where adjustments to proton therapy may be warranted.

## 2 MATERIALS AND METHODS

### 2.1 Study design

This retrospective cohort study was conducted at Siteman Cancer Center, involving HN, breast/CW and prostate patients who received pencil‐beam‐scanning proton therapy (PBS‐PT) between January 1, 2020, and April 15, 2023, after obtaining ethical approval.

In total, 166 HN, breast/CW, and prostate patients were treated with PBS‐PT during this time period. Of these, 117 patients, representing all three groups (60 HN, 34 breast/CW, and 23 prostate cases), had in‐room CT on‐rails (CToR) scans (SOMATOM Definition Edge, Siemens Healthineers, Erlangen, Germany) during their treatment course, resulting in a total of 890 scans. Considering the clinical evaluations and physician decisions, patients who did not undergo CToR scanning during treatment were supposed to be stable within the treatment, eliminating the need for follow‐up CToR scans.

Planning data were extracted from Aria (Varian Medical System) and RayStation (RaySearch Laboratories) and included details regarding treatment site, overall fraction count, total number of CToR scans, fraction number of replanning, and the rationale behind replanning.

The original plans were generated according to clinical guidelines for each disease site. HN cases were planned with two to four fields depending on target size and location, and optimized with multi field optimization (MFO). While the plans for all patients were robustly optimized, for some cases the plan was optimized based on clinical target volume (CTV) with 3–5 mm setup uncertainty and 3% range uncertainty, whereas for others, the plan was optimized based on planning target volume (PTV) and a 2% range uncertainty. Breast plans typically involved two to four fields with MFO and a 2% range uncertainty to achieve robust PTV coverage. Prostate plans were PTV base and employed two opposed lateral fields with single‐field optimization (SFO), with each field delivering approximately half of the prescription dose and robust optimization performed with a 2% range uncertainty. Criteria for replanning and adaptation are summarized in Tables 1 and 2.

**TABLE 1:** Head and neck replanned proton therapy cases‐diagnosis, fraction number of replanning, DVH indices, and reason for replanning.

					Dosimetric evaluation	
Case	Diagnosis	Number of replans	Fraction number of replanning	Rationale	Constraint	evaluated
1	Malignant neoplasm of dorsal surface of tongue	2	11/35	External anatomy changes	CTV___7000 ≥ 95%	68.8%
			16/35	External anatomy changes	CTV_7000 ≥ 95%	70%
2	Malignant neoplasm of tonsil, unspecified	1	16/20	Setup uncertainty (non‐reproducibility between planning CT and CToR due to patient setup)	PTV___6000 ≥ 95%	86.5%
3	Malignant neoplasm of tonsil, unspecified	1	17/30		External anatomy change PTV___6000 ≥ 95%	87.8%
4	Secondary malignant neoplasm of other specified sites	2	16/35	Tumor deformation (growth)	PTV___7000 ≥ 95%	84.4%
			27/35	Tumor deformation (shrinkage)	PTV_7000 ≥ 95%	51.0%
5	Secondary and unspecified malignant neoplasm of lymph nodes of head, face, and neck	1	2/31	External anatomy changes	PTV_6600 ≥ 95%	81.2%
6	Malignant neoplasm of the connective and soft tissue of the head, face, and neck	1	2/31	External anatomy changes	CTV___5580≥ 95%	71.5%
7	Malignant neoplasm of maxillary sinus	1	12/25	Internal and external anatomy changes	Brain Stem _D _max_ < 5400 cGy	6100 cGy
8	Malignant neoplasm of the pharynx, unspecified	1	12/33	Setup uncertainty	PTV_6600 ≥ 95%	79.9%

**TABLE 2:** Breast and chest wall replanned proton therapy cases‐diagnosis, fraction number of replanning, DVH indices, and reason for replanning.

					Dosimetric evaluation	
Case	Diagnosis	Replanning number	Fraction number of replanning	Rationale	Prescribed	Evaluated
1	Left breast lower outer quadrant carcinoma	1	6/16	External anatomy changed (silicone implant added)	PTV_4256 ≥ 95%	89.2%
2	Left breast overlapping‐site carcinoma	1	4/16	Metal magnet implant displacement	PTV_4256 ≥ 95%	8.8%
3	Left breast lower outer quadrant carcinoma	1	4/16	Metal magnet implant displacement	PTV_4256 ≥ 95%	70.4%
4	Left breast lower inner quadrant carcinoma	1	5/25	Metal magnet implant displacement	PTV_5000 ≥ 95%	88.4%
5	Left breast overlapping‐site carcinoma	1	3/16	OAR overdose (Lung)	V_15%_ < 2000 cGy	2566 cGy
6	Right breast overlapping‐site carcinoma	3	(6, 14, 17)/25	Metal magnet implant displacement (×2)	PTV_5000 ≥ 95%	82.7%
						76.7%
				External body contour change	PTV_5000 ≥ 95%	70.8%

### 2.2 Replanning investigation in Raystation

**FIGURE 1:**
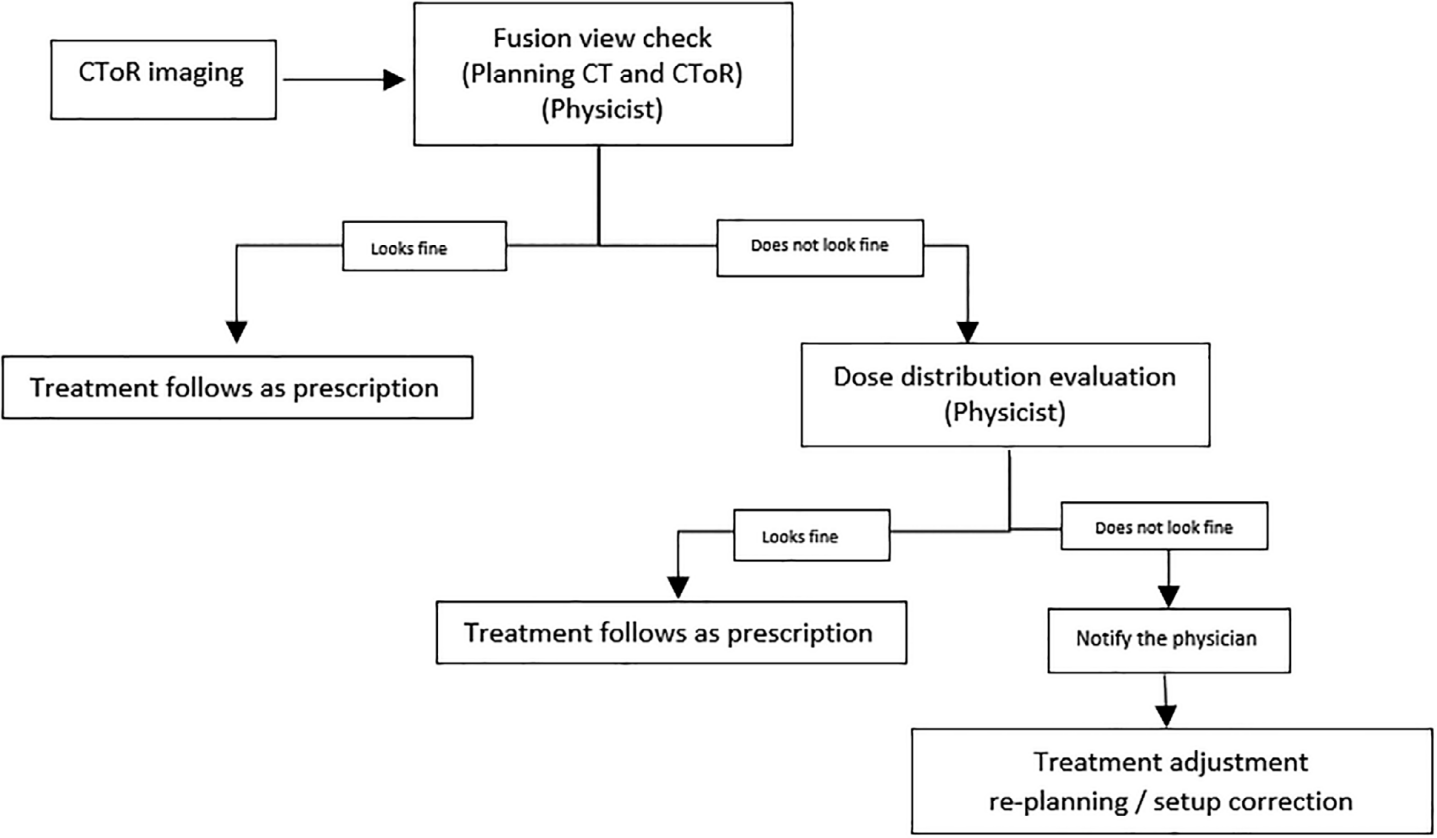
Workflow of the offline adaptive proton therapy process. Following CToR imaging, the acquired dataset is fused with the planning CT by a physicist. If the fusion check indicates that the anatomy is acceptable, the treatment proceeds as prescribed. If discrepancies are observed, the dose distribution is evaluated. When the evaluated dose distribution remains clinically acceptable, treatment continues as prescribed. If the dose distribution is unacceptable, the physician is notified and a decision on the method of adaptation is made. Treatment adaptation is then performed, which may involve re‐planning or setup correction prior to continuation of therapy.

The process for implementing replanning during actual treatment (between 2020 and 2023) is detailed in Figure 1. If any suspicious anatomical changes or setup uncertainty were noted within the fusion view of the planning CT and the CToR of the day for the patient, a dose evaluation was triggered. This evaluation included multiple steps such as image registration, contour refinement, and dose recalculation on the CToR of the day in RayStation (RS). Using the image registration capabilities of the RS system, contours initially drawn on the planning CT for the primary treatment plan were transferred to CToR using a combination of rigid and deformable registration methods: target contours were rigidly transferred and if there were significant anatomical differences in the target region, they were edited as needed. For OARs, contours were deformably transferred, and the propagated contours were then adjusted. The treatment plan was recalculated on the CToR scans and evaluated. When indicated (dose deviation for target or critical organs), treatment adjustments including setup corrections via patient repositioning or replanning, were made based on this evaluation.

For the plans which had been adapted during the treatment course, the rationale for replanning was determined by assessing several factors including the changes in anatomy, dose distribution, and dose‐volume histogram (DVH) between the initial simulation CT scans and the CToR scans. Setup inconsistencies in internal and external anatomy that justified replanning were detected by the misalignment of anatomy between the initial planning CT and the CToRs. Dosimetric justification for replanning was assessed by evaluating dose coverage in the treatment target and OARs of high priority (optic nerves, cochlea, spinal cord, brain stem, lungs and etc.), together with various DVH metrics. In prostate cases, target coverage was the main consideration for replanning by physicians.

## 3 RESULTS

### 3.1 Frequency of CToR acquisition and replanning

**FIGURE 2:**
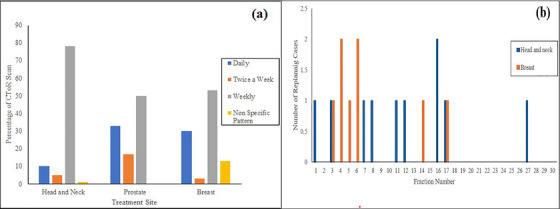
(a) CToR imaging frequency for head and neck, prostate, and breast cases. (b) Replanned fractions in head and neck, and breast cases.

CToR acquisition was obtained weekly for the majority of cases, followed by daily, and bi‐weekly (Figure 2a). Some cases involved CToR acquisition at non‐specific pattern at the discretion of treating radiation oncologist. Replanning was required in 11% of all cases, with the highest percentage observed in breast/CW cases (16%) and followed by HN cases (13%). None of the prostate cases required replanning. Figure 2b illustrates the replanning frequency for breast/CW and HN cases. In the breast/CW cases with treatment courses of 16 to 25 fractions, replanning was more frequent at fractions 3–6 and 14–17, yet the limited cohort size suggests caution in interpreting these findings as indicative of a complete absence of replanning at other intervals. In HN cases with treatment course ranging from 20 to 33 fractions, there did not appear to be a pattern in the identification of fractions that trigger replanning, as shown in Figure 2b.

### 3.2 Rationale for replanning

**FIGURE 3:**
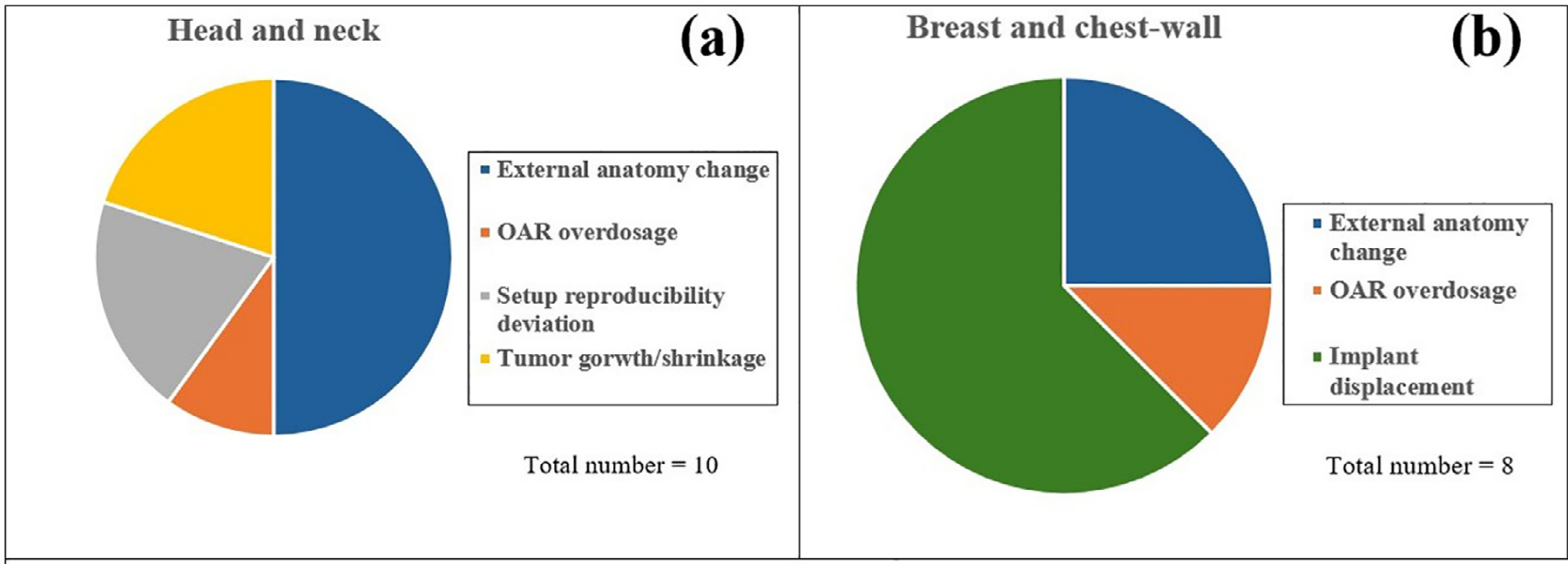
Distribution of replanning reasons in head and neck and breast/chest‐wall (CW) cases.

Replanning decisions were made by evaluating both target coverage and OAR sparing. In HN cases (Figure 3a and Table 1), the reasons for replanning were predominantly associated with a loss of target coverage, which was primarily attributed to external anatomy changes resulting from weight changes in five cases, tumor growth/shrinkage in two cases, and set‐up uncertainty in two cases, while replanning for OAR over‐dosage was identified only in one specific case, which originated from internal anatomy changes.

In breast/CW cases (Figure 3b and Table 2), the decision to replan was based on the displacement of tissue expander magnets five times (6 out of 50 patients had metal magnet implants), and changes in the external body contour led to replanning in two cases. Both scenarios resulted in a target coverage compromise. OAR over‐dosage was the primary reason for replanning in one case.

### 3.3 HN

Table 1 lists the rationale of replanning for eight HN cases (10 times), as well as diagnosis, frequency of replanning, fraction number for new plan, and DVH parameters.

**FIGURE 4:**
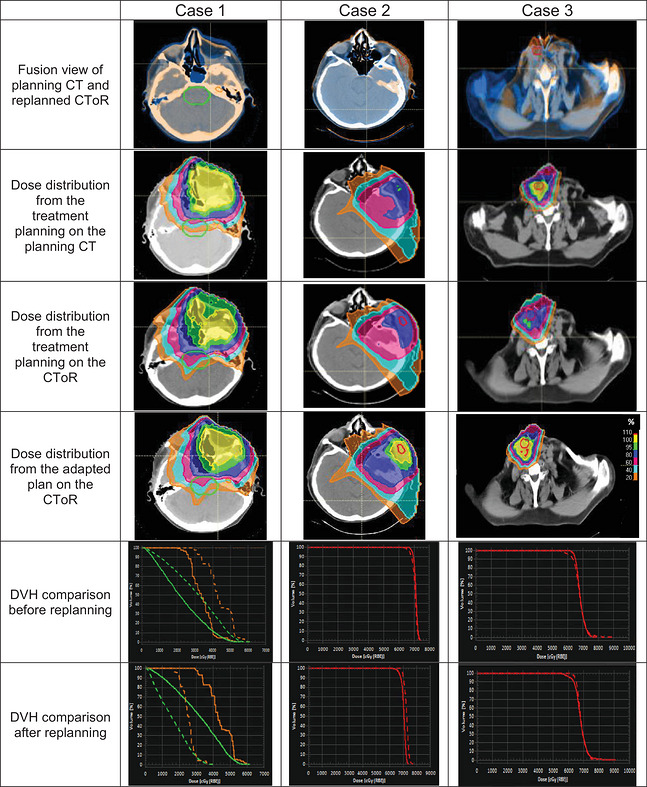
Visual representation of three distinct head and neck cases illustrating the impact of anatomical changes on treatment planning. Case 1 illustrates example of anatomy change where clearing of the nasal cavity contents and the reduction of swelling on the left side of the face inadvertently led to overdosing of critical OARs including brainstem (green contour) and cochlea (orange contour). Case 2 illustrates tumor growth during the treatment course, necessitating redefinition of the gross tumor volume (GTV), shown as a red dashed line in the fusion view and as a solid line in the evaluation and adjusted plan. Case 3 depicts an example of setup irreproducibility requiring replanning. In this case, positioning changes causing an anterior shift in the GTV (the red contour) resulted in compromise of GTV coverage. DVH of each case before and after replanning that results in optimal target coverage and OAR sparing are shown in the two last rows. The solid lines in the first row of DVH comparison (DVH before replanning) are representative of planning CT and dashed line are related to evaluated CToR, while in the second row (DVH after replanning), the dashed line corresponds to the replanning evaluation results, and the solid line signifies the evaluation of the primary planning on the same CToR.

The rationale for replanning for HN cases fell under 3 categories: setup uncertainty, target changes and anatomy changes (internal and external). Figure 4 visually demonstrates an example of each category.

### 3.4 Breast and chest wall

**FIGURE 5:**
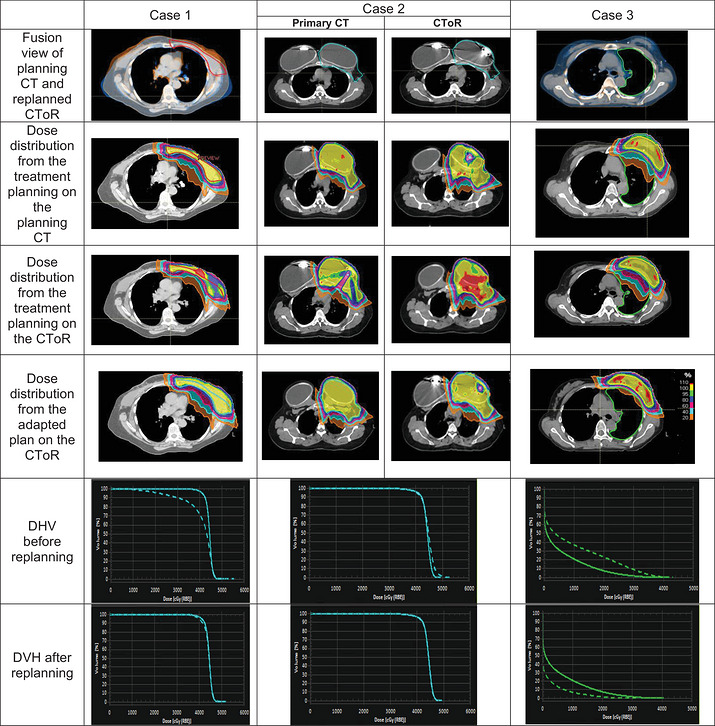
Visual representation of three distinct breast and chest wall cases illustrating the impact of external anatomical changes (Case1), metal implant displacement (Case2) and OAR overdosage (Case3) on treatment planning. For Case 1, the dose distribution and DVHs indicate the need for plan adaptation due to PTV under‐dosage. In case 2, a shift in the position of the tissue expander magnet resulted in change of the planned dose distribution, requiring replanning with the implant's new position for consistent conformity and coverage (for easier tracking of the metal magnet implant's movement, the planning CT and CToR are shown separately in this case). The third case highlights OAR overdose to the lung (green contour), with replanning adjusting beam angles to tangential to reduce lung dose while maintaining PTV coverage.

Rationale for replanning in breast/CW cases fell under 3 different categories: displacement of tissue expander magnet, anatomy change, and OAR over‐dosage. Figure 5 visually demonstrates an example of each category.

### 3.5 Prostate

**FIGURE 6:**
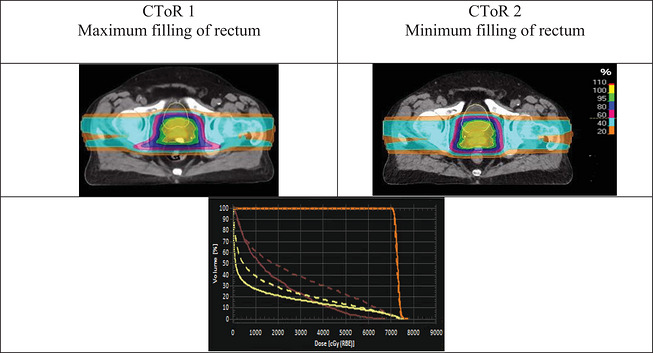
Visual representation of a prostate case with different filling of bladder (yellow contour) and rectum (brown contour). CToR 1 maximum filling, CToR 2 minimum filling. The related DVHs are shown in the third row: solid lines correspond to the planning CT, and dashed lines correspond to the evaluation on CToR with different bladder (yellow) and rectum (brown) fillings, as well as the prostate DVH (orange).

**FIGURE 7:**
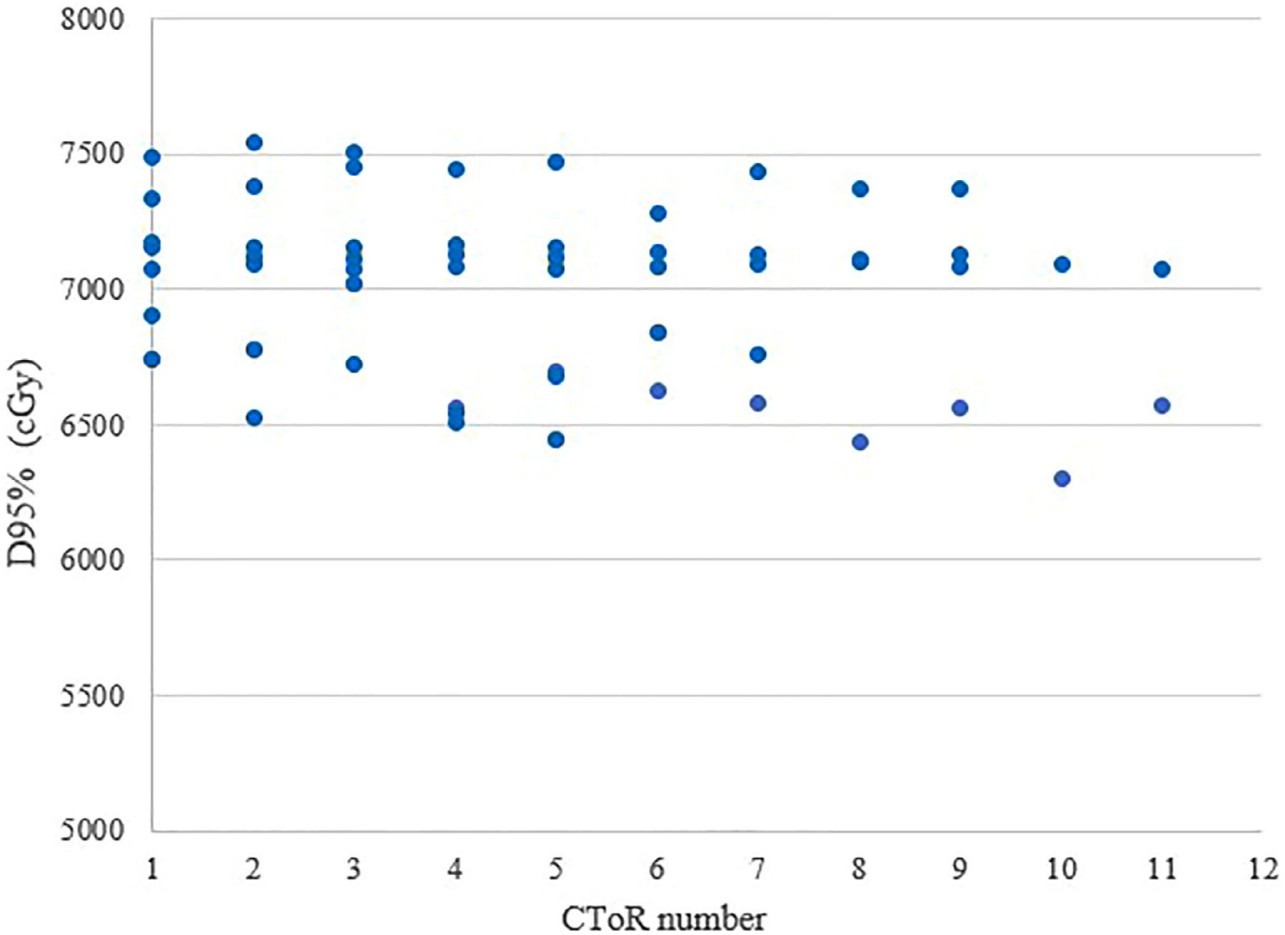
D95% variation of some of the prostate PTVs across different CToRs.

Our study demonstrates that despite the frequent observation of anatomical changes, including variations in patient weight and bladder/rectum filling, none of the prostate patients evaluated underwent replanning. Figure 6 illustrates a case where there was a significant difference in the bladder and rectum volume between planning CT and daily CToRs. Corresponding DVHs show that bladder and rectum filling variations did not significantly impact target coverage. The result of PTV coverage (D_95%_) evaluations during the treatment sessions (Figure 7) indicated consistent coverage for prostate cases.

## 4 DISCUSSION

**FIGURE 8:**
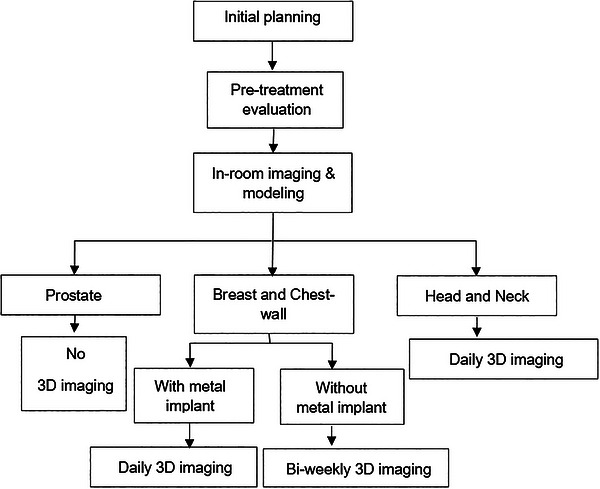
Suggested workflow for 3D imaging (CToR or CBCT) frequency in head and neck, breast/chest wall, and prostate cases.

In this large retrospective review of our patient, the rationale and frequency for offline adaptation in proton therapy for HN, breast/CW, and prostate cancer patients were characterized. For HN patients receiving proton therapy, it was observed that 8 out of the 76 cases required replanning, with 2 out of the 8 cases being replanned twice (13% replanning in total). Most of these cases (7 out of 8) necessitated offline adaptation due to changes in target coverage resulting from external anatomy changes, tumor growth, and setup uncertainty. We did not identify a specific pattern regarding when offline adaptation occurred most frequently. In a comparable study, Ger et al. reported that 25% of HN patients required adaptation and recommended weekly quality assurance CT (QACT).^10^ In contrast, our findings suggest that adaptation may be necessary at any treatment fraction, supporting the use of daily imaging as indicated in Figure 8. The main distinction between the two studies lies in the imaging approach: Ger et al. utilized CBCT, while our analysis was based on daily CToR imaging. The ability to perform daily dose calculations using CT data likely increases the sensitivity for detecting when adaptation is needed. Studies with smaller cohorts such as Yang et al. reported that 40% (4 out of 10 cases) of HN patients treated with intensity‐modulated proton therapy (IMPT) required at least one instance of adaptation.^11^ Similarly, in‐silico small cohort studies have reported inadequate target coverage in 25–60% of HN cases treated with IMPT due of anatomical changes.^12^, ^13^ In a review by Huiskes et al., offline adaptation was mostly utilized in HN patients treated with proton therapy in order to improve target coverage.^9^ Notably, they did not identify a particular timing pattern for these offline adaptations. Narita et al. specifically examined maxillary sinus carcinoma treated with protons and observed that offline adaptation for OARs protection typically occurred around 3–4 weeks into treatment.^14^ Similarly, Scandurra et al. reported 2 out of 25 patients with nasopharyngeal carcinoma needed plan adjustments due to reduced target coverage with offline adapted plan satisfying dose constraints to critical OARs.^15^ Although our overall adaptation rate of 13% appears lower, it is important to note that the current study considered a more diverse spectrum of HN cases, with variations in clinical indications. The discrepancies between the findings highlight the complexity of plan adaptation needs and the diverse challenges faced in the field of HN proton therapy.

In the examination of 50 cases involving the breast/CW, 6 cases required offline adaptation, and one case was replanned three times. Five of the eight (63%) offline adaptations were due to tissue expander magnet migration while 25% (2 out of 8) were related to external anatomy changes. This closely aligns the findings by Ger et al. where it was noted that 60% of cases requiring replanning in breast cases were attributed to the displacement of tissue expander magnets while 40% were attributed to other reasons that were not specifically defined.^8^ Additionally, another study by Jensen et al. focused on planning and adaptation strategies for breast/CW cases treated with protons and emphasized the importance of addressing anatomical variations, such as tumor cavity shrinkage and swelling.^16^ Based on our clinical experience, breast cases with implants are recommended to undergo daily CToR imaging, or at minimum, 3D imaging for precise monitoring, whereas breast and chest wall cases without reconstruction are recommended to have at least two QACT sessions during the treatment course to ensure accuracy and enable adaptive adjustments, as shown in Figure 8. In breast/CW cases, the movement of tissue expander magnets presents a unique challenge as shifts in magnet position can result in significant changes in the dose distribution, potentially causing unintended variations in dose delivery within the treatment area or overdosing of nearby OARs, particularly the heart and lungs.

For prostate cancer treated with protons, we did not find any cases where offline adaptation was necessary. In all cases, PTV coverage remained consistent within an acceptable range without requiring adaptation. However, incorporating a broader range of scenarios into the uncertainty distributions used for robust planning could help ensure adequate coverage in cases with greater anatomical variations or setup uncertainties.^17^ In a study by Tamura et al. the benefits of daily adaptive radiotherapy for high‐risk prostate cancer, focusing on the CTV, prostate, seminal vesicle, rectum, and bladder were explored.^6^ Their study identified significant differences in accumulated doses for CTV and seminal vesicle between non‐adaptive and daily adaptive radiotherapy, but no significant variations in doses to the prostate, rectum, and bladder. It is essential to note that these differences in the outcomes of these studies may stem from distinct research approaches. The current study was retrospective, prioritizing tumor coverage, while the study by Tamura et al took a retrospective approach with an emphasis on safeguarding OARs. Variations in planning approaches among different centers may contribute to differences in tumor dose coverage and the necessity for plan adaptation.

The strengths of this study include a large cohort of HN, breast/CW, and prostate patients receiving proton radiation therapy with CToR imaging available in‐room during their treatment course. The limitations of the study include the retrospective nature of the analysis and the varying intervals of CToR acquisition as dictated by the treating physician.

## 5 CONCLUSION

Our analysis of patients undergoing proton therapy for HN, breast/CW, and prostate cancers revealed that anatomical changes affecting target coverage, OAR sparing, and setup accuracy were the primary reasons for offline adaptation. HN cases showed a recurring need for plan adaptation throughout the treatment course, reflecting the complex and dynamic anatomy in this region. Among breast/CW cases, those with implants demonstrated a higher susceptibility to plan adaptations, often from the beginning of the treatment. In contrast, prostate cases were the most robust, maintaining consistent target coverage despite variations in bladder and rectal filling. This study enhances our understanding of clinical scenarios that may necessitate adaptation, helping us remain well‐prepared and responsive when such situations arise.

## AUTHOR CONTRIBUTIONS

**Yao Hao**: Conceptualization, methodology, data collection, writing–review and editing. **Eric Laugeman**: Conceptualization, data collection, analysis, writing–review and editing. **Weiren Liu**: Methodology, analysis, writing–original draft, writing–review and editing. **Farideh Pak**: Conceptualization, methodology, data collection, analysis, writing–original draft, writing–review and editing. **Stephanie Perkins**: Conceptualization, methodology, writing–review and editing. **Joshua Schiff**: Conceptualization, analysis, writing–review and editing. **Matthew Schmidt**: Methodology, data collection, writing–review and editing. **Michael Watts**: Conceptualization, methodology, data collection, writing–review and editing. **Hailei Zhang**: Data collection, analysis, writing–review and editing. **Tianyu Zhao**: Conceptualization, methodology, analysis, writing–original draft, writing–review and editing.

## FUNDING INFORMATION

This work was supported by the Kling Professorship.

## CONFLICT OF INTEREST STATEMENT

The authors declare no conflicts of interest.

## ETHICAL APPROVAL

This study was approved by the Institutional Review Board (IRB ID: 202305134).

## Data Availability

All data generated or analyzed during this study are included in this published article.
