# Correction to “Retrospective analysis of adaptation frequencies and factors in offline in offline adaptive proton therapy”

**DOI:** 10.1002/acm2.70796

**Published:** 2026-09-29

Pak F, Zhang H, Hao Y, et al. Retrospective analysis of adaptation frequencies and factors in offline in offline adaptive proton therapy. *J Appl Clin Med Phys*. 2026;27:e70604. https://doi.org/10.1002/acm2.70604

We noticed a typographical error in the final published title where a word (in offline) is accidentally repeated.

- Current Title: Retrospective analysis of adaptation frequencies and factors in offline in offline adaptive proton therapy
- Corrected Title: Retrospective analysis of adaptation frequencies and factors in offline adaptive proton therapy

We apologize for this error. The online version of this article has been corrected accordingly.

